# Underpinning Sustainable Vector Control through Informed Insecticide Resistance Management

**DOI:** 10.1371/journal.pone.0099822

**Published:** 2014-06-16

**Authors:** Edward K. Thomsen, Clare Strode, Kay Hemmings, Angela J. Hughes, Emmanuel Chanda, Mulenga Musapa, Mulakwa Kamuliwo, Faustina N. Phiri, Lucy Muzia, Javan Chanda, Alister Kandyata, Brian Chirwa, Kathleen Poer, Janet Hemingway, Charles S. Wondji, Hilary Ranson, Michael Coleman

**Affiliations:** 1 Vector Biology Department, Liverpool School of Tropical Medicine, Liverpool, United Kingdom; 2 National Malaria Control Centre, Lusaka, Zambia; 3 Zambia Integrated Systems Strengthening Program, Abt Associates, Lusaka, Zambia; University of Notre Dame, United States of America

## Abstract

**Background:**

There has been rapid scale-up of malaria vector control in the last ten years. Both of the primary control strategies, long-lasting pyrethroid treated nets and indoor residual spraying, rely on the use of a limited number of insecticides. Insecticide resistance, as measured by bioassay, has rapidly increased in prevalence and has come to the forefront as an issue that needs to be addressed to maintain the sustainability of malaria control and the drive to elimination. Zambia's programme reported high levels of resistance to the insecticides it used in 2010, and, as a result, increased its investment in resistance monitoring to support informed resistance management decisions.

**Methodology/Principal Findings:**

A country-wide survey on insecticide resistance in Zambian malaria vectors was performed using WHO bioassays to detect resistant phenotypes. Molecular techniques were used to detect target-site mutations and microarray to detect metabolic resistance mechanisms. *Anopheles gambiae s.s.* was resistant to pyrethroids, DDT and carbamates, with potential organophosphate resistance in one population. The resistant phenotypes were conferred by both target-site and metabolic mechanisms. *Anopheles funestus s.s.* was largely resistant to pyrethroids and carbamates, with potential resistance to DDT in two locations. The resistant phenotypes were conferred by elevated levels of cytochrome p450s.

**Conclusions/Significance:**

Currently, the Zambia National Malaria Control Centre is using these results to inform their vector control strategy. The methods employed here can serve as a template to all malaria-endemic countries striving to create a sustainable insecticide resistance management plan.

## Introduction

Significant headway has been made within the last decade in the global fight against malaria [1] with some countries now entering the malaria elimination phase [2]. The primary tools used to reduce malaria burden have been vector control and improved case management. As a result, 274 million cases and 1.1 million deaths from malaria have been averted between 2001 and 2010 [3] through the distribution of insecticide treated nets (ITN), the implementation of indoor residual spraying (IRS) of insecticide, and the utilization of artemisinin combination therapy with improved diagnostic capabilities. ITNs and IRS continue to be the pillars of most national malaria control programmes. The percent of households owning at least one ITN has increased across malaria endemic regions of Africa from 3% in 2000 to 53% in 2012, and the number of people covered by IRS has more than doubled since 2005 [3].

However, the development of insecticide resistance threatens to compromise these gains. In South Africa, malaria cases quadrupled four years after the introduction of pyrethroids for IRS in 1996. The *Anopheles funestus* population was resistant to pyrethroids and was able to re-establish itself having been eliminated from the country [4]. With the reintroduction of DDT in 2001, *An. funestus* was again controlled and malaria cases declined by 91%. [5]. Another study in Senegal documented resurgence in malaria incidence to pre-intervention levels just 2.5 years after the introduction of long-lasting insecticidal nets (LLINs). The authors suggested that a significant increase in a point mutation that confers resistance in the vector population contributed to control failure [6]. In experimental hut trials in Benin, pyrethroid-based vector control (either impregnated into LLINs or sprayed onto walls) was significantly less effective in inhibiting blood-feeding and killing mosquitoes in areas with pyrethroid-resistant populations than in areas with pyrethroid-susceptible populations [7]. A predominantly vector-centric control strategy coupled with increasing levels of insecticide resistance poses a significant challenge to the global malaria elimination community [8]. As such, it is vital to establish a surveillance system to monitor emerging resistance and mitigate its effects [9].

Zambia has been a leader in sub-Saharan Africa in implementing an ambitious malaria control programme [10], [11]. With targets of universal coverage of vector control and a 75% reduction in malaria incidence between 2010 and 2015 [12], the country's ambitions largely surpass those set by the Roll Back Malaria Partnership [13]. In 2012, 73% of households either had at least one ITN or had been protected with IRS [14]. This, in concert with improved treatment, diagnosis, and intermittent preventative treatment in pregnancy (IPTp) led to a reduction in malaria mortality by 66% between 2001 and 2009 [15].

However, WHO bioassays were completed in 2010 and detected insecticide resistance to 3 of the 4 insecticide classes recommended by the World Health Organization (WHO) for IRS [16]. Initial geographic coverage of resistance data was limited to nine districts in three provinces surrounding the capital of Lusaka. IRS expanded to 54 districts in 2010 and all 72 districts in 2011 [17]. With control measures rapidly scaling up, insecticide resistance confirmed, and a lack of resistance data in much of the country, the potential for control failure was clear. This prompted the establishment of a national insecticide resistance management technical working group and enhanced efforts to monitor insecticide resistance and the mechanisms present in the country. Here we report the data generated from these efforts and discuss the implications for future malaria vector control.

## Methods

### Study Sites

Zambia is located in the Southern African region with a population of over 13 million [18], and malaria is endemic throughout the country [12]. The study sites for entomological monitoring are distributed nation-wide. They were selected to assist the expanding vector control programme and to provide evidence for informed decision-making.

**Figure 1:**
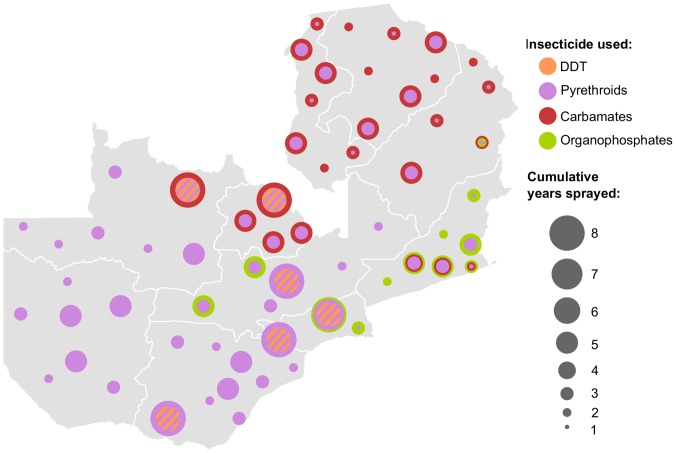
Spatiotemporal pattern of insecticide use for IRS in Zambia from 2005–2012. Each dot represents the insecticide history for a single district or cluster of districts with similar history (Copperbelt Province). The earliest insecticide used is indicated in the centre of each dot. Subsequent insecticides are added as layers, with the thickness of the layer representing how many years the insecticide was used. Different colours represent different insecticide classes. The size of the dot indicates how many years IRS has been active. Hashed areas indicate times and locations where DDT and pyrethroids were used concurrently, with the former on mud homes and the latter on painted surfaces.

Countrywide mass distribution of ITNs started in 2005, and currently 72% of households own at least one net [14]. Since 2007, only LLINs (either Permanet from Vestergaard Frandsen, Netprotect from BestNet, or Olyset from Sumitommo) have been distributed [10]. Prior to 2005, IRS was conducted primarily in Copperbelt Province surrounding mining communities [19]. IRS was scaled up to include 15 districts in 2005, 36 in 2008, 54 in 2010, and 72 in 2011 [11], [17]. Until 2007, spraying was targeted in urban and peri-urban zones, but since then it has expanded to more rural areas to better align the intervention with malaria burden [14]. DDT or pyrethroids were sprayed in the original 15 districts from 2005–2010. Districts added in 2008 and 2010 were sprayed with pyrethroids (λ-cyhalothrin, deltamethrin, or alpha-cypermethrin; [20]). The insecticides used in each area of the country were modified in 2011 (figure 1). Spraying occurs in October-December of each year, prior to the peak transmission season.

### Mosquito collections

Blood-fed and gravid adult female mosquitoes were collected resting inside on the walls of private dwellings between 0400–0900 hrs using a modified CDC backpack aspirator from March 2011 to April 2013 from 54 localities in 26 districts (Table S1). Verbal consent was obtained from the home owner before collections began. This study did not involve endangered or protected species. Anopheline mosquitoes were identified morphologically as *An. gambiae* Giles complex or *An. funestus* Giles group [21], [22]. Mosquitoes were induced to lay eggs in individual oviposition tubes. Eggs were either transported to the Liverpool School of Tropical Medicine (LSTM) or reared at the National Malaria Control Centre (NMCC) in Lusaka, Zambia. Egg batches from females collected at each locality were pooled and reared together to avoid bias from isofemale lines. F0 females were preserved and sent to LSTM for sibling species identification by PCR [23]–[25].

### Insecticide resistance bioassays

Insecticide resistance bioassays were carried out on a random sample of 2–5 day old, sugar fed F1 adults following the standard procedure described by the WHO [26]. Both male and female mosquitoes were exposed to insecticide (or control papers) for 60 minutes, and allowed to recover with access to 10% sucrose solution for 24 hours before recording the percentage mortality. Insecticides tested included bendiocarb (0.01%), DDT (4%), deltamethrin (0.05%), etofenprox (0.5%), λ-cyhalothrin (0.05%), malathion (5%) permethrin (0.75%), pirimiphos-methyl (0.25%) and propoxur (0.1%). Pirimiphos-methyl papers were made by diluting the discriminating concentration [26] in acetone and impregnating filter paper. All other papers were purchased from the WHO.

The entomological and mapping tools of the Disease Data Management System [27] were used to manage the data. The mortality from all bioassays in which control mortality was 5–20% was corrected using Abbott's formula [28]. All assays performed on mosquitoes from a single district were aggregated over the course of a year. 95% confidence intervals were calculated using Wilson's method with continuity correction [29]. Populations were classified as resistant if there was less than 90% mortality, potentially resistant if mortality was between 90–98%, and susceptible if mortality was greater than 98% [26]. Any comparisons between mortality rates were performed using χ^2^ tests.

### Resistance mechanisms

PCR assays were carried out to detect target-site mediated resistance. The presence of both the east (1014S) and west (1014F) *kdr* alleles of the voltage-gated sodium channel gene [30] and the insensitive allele (119S) of the acetylcholinesterase (*iACE*) gene [31] was investigated in a random sample of *An. gambiae s.s.* F0 females.

Genome-wide transcriptional analysis using microarrays was used to detect metabolically mediated resistance in 6 districts. Total RNA extractions were performed on pools of 30 non-blood fed 5 day old female mosquitoes which had either survived insecticide exposure (R), were wild but unexposed (C), or were from laboratory susceptible strains (S – Kisumu for *An. gambiae* and FANG for *An. funestus*). Mosquitoes from Kitwe (Copperbelt Province) and Kasama (Northern Province) districts were exposed to deltamethrin, Katete (Eastern Province) and Luangwa (Lusaka Province) to etofenprox, and Kaoma (Western Province) to λ-cyhalothrin. Unexposed mosquitoes were extracted from Luangwa, Kaoma, and Solwezi (North-western Province). Four separate extractions served as biological replicates. RNA extractions were performed using PicoPure extraction kit (Arcturus) according to the manufacturer's instructions and DNase treated (RNAse-free DNAse kit, Qiagen). The quality and quantity of the RNA in the combined pools were assessed using a Bioanalyzer (Agilent) and a NanoDrop spectrophotometer (NanoDrop Technologies), respectively.

RNA pools selected for microarray analysis were labelled separately with Cy3 and Cy5 dyes using the Low Input Quick Amp Labeling Kit (Agilent). The quantity and quality of the labelled RNA samples were assessed as described above. Only samples that passed Agilent's recommendations for >825 ng yield and specific activity greater than 6.0 pmol of cynanine (Cy) per microgram of cRNA were used on the microarray.

*An. gambiae s.s.* populations were hybridised using a custom ‘AGAM_15K' platform (ArrayExpress accession number A-MEXP-2196) [32] and *An. funestus s.s.* populations used a custom designed ‘AFUN_60K' platform. Array hybridization, washing, scanning, and feature extraction were performed according to the manufacturer's recommendations. Microarray normalization using locally weight scatterplot smoothing was performed during feature extraction. Normalised data were analysed using GeneSpring v.12 software (Agilent). In brief, data was subjected to a student's t-test with post-hoc correction of the p-value using Benjamini-Hochberg False Discovery Rate. Genes were considered differentially expressed if they presented a ±2 fold change (FC) in expression level between the susceptible and resistant populations alongside a corrected p-value <0.05.

Selected microarray data from the six districts were validated using quantitative reverse transcriptase PCR (qRT PCR, primers available in Table S2). Two additional districts without microarray data were analysed as well. cDNA was synthesised from total RNA from the four biological replicates used in the microarray study using SuperScript III (Invitrogen) according to the manufacturer's instructions. qRT PCR was performed using 10 µM of each primer and 10 ng cDNA in a 20 µL reaction volume using Brilliant III Ultra-Fast SYBR Green qPCR Master Mix (Agilent). qRT PCR amplification was performed using a MX 3005 real-time PCR system (Agilent) with the following program: denaturation  = 95°C for 3 mins, 40 cycles  = 10 secs at 95°C, 10 secs at 60°C, final step  = 1 min at 95°C, 30 secs at 55°C and 30 secs at 95°C. Serial dilutions of cDNA were used to create standard curves for each gene in order to assess PCR efficiency and quantitative differences between the samples. Relative gene expression and associated FC between samples was quantified using the 2^−ΔΔCT^ method [33] after normalisation to the appropriate control genes (S7 and elongation factor for *An. gambiae s.s.* and S7 and tubulin/actin for *An. funestus s.s.*) and incorporating PCR efficiency. Relative 2^−ΔΔCT^ values were compared between populations using t-tests.

### Sporozoite detection

DNA was extracted from the head and thorax of wild caught F0 mosquitoes after laying eggs, and tested for the presence of *Plasmodium* spp. sporozoites [34].

## Results

### Mosquito Collections

Seventy-three wild *An. gambiae s.l.* and 421 wild *An. funestus s.l.* from 7 provinces were confirmed to species with PCR after laying eggs. All *An. gambiae s.l.* were confirmed as *An. gambiae s.s.* and all *An. funestus s.l.* were confirmed as *An. funestus s.s.*

### Insecticide resistant phenotypes

A total of 3097 *An. gambiae s.s.* and 5806 *An. funestus s.s.* were assayed for resistant phenotypes between March 2011 and April 2012. An additional 3374 and 1461, respectively, were assayed between May 2012 and April 2013. Families came from a total of 26 districts representing all 10 provinces (Muchinga Province was recently added in 2011).

**Figure 2:**
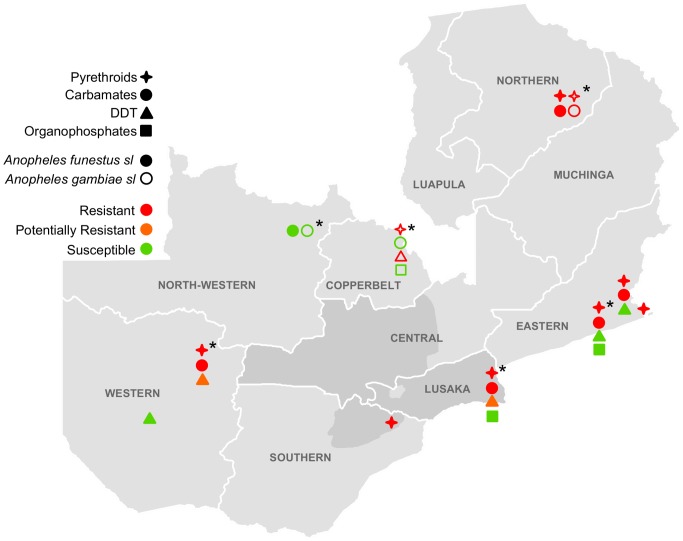
Insecticide resistance in collections from March 2011–April 2012. Darker gray shading indicates areas surveyed in [16]. *locations with microarray data.

**Figure 3:**
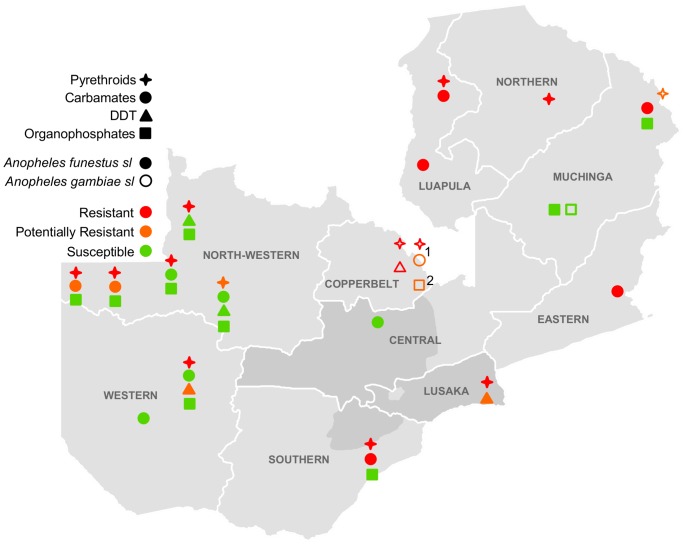
Insecticide resistance in collections from May 2012–April 2013. Darker gray shading indicates areas surveyed in [16]. ^1^Potentially resistant to bendiocarb but susceptible to propoxur. ^2^Potentially resistant to pirimiphos-methyl but susceptible to malathion.

*An. gambiae s.s.* was resistant to DDT and pyrethroids throughout its range. Mortality to deltamethrin ranged from 39–83% in 2011–2012 and 65–94% in 2012–2013 (one population was potentially resistant in Isoka District). Carbamate resistance was detected in one location in 2011–2012 (Kasama District) and potential carbamate resistance was detected in a separate location in 2012–2013 (Masaiti District). Potential organophosphate resistance was detected in 2012–2013 from Masaiti as well (Tables 1 and 2, Figures 2 and 3).

**Table 1:** WHO bioassay test results on 2–5 day old adult mosquitoes from March 2011-April 2012 from 14 districts in Zambia.

Location															
Province	District	Deltamethrin (0.05%)		Permethrin (0.75%)		λ-cyhalothrin (0.05%)		Etofenprox (0.50%)		DDT (4%)		Malathion (5%)		Bendiocarb (0.01%)	
		n	% mortality (95%CI)	n	% mortality (95%CI)	n	% mortality (95%CI)	n	% mortality (95%CI)	n	% mortality (95%CI)	n	% mortality (95%CI)	n	% mortality (95%CI)
***An. funestus s.l.***															
North-Western	Solwezi													20	100 (80.0, 100)
Western	Kaoma	180	75.6 (68.5, 81.5)	24	100 (82.8, 100)	738	31.3 (28.0, 34.8)	119	10.1 (5.6, 17.3)	273	93.4 (89.6, 95.9)			330	60.6 (55.1, 65.9)
	Senanga									30	100 (85.9, 100)				
Lusaka	Luangwa	108	42.6 (33.2, 52.5)			37	64.9 (47.4, 79.3)	454	29.5 (25.4, 34.0)	190	97.4 (93.6, 99.0)	101	100 (95.4, 100)	370	34.6 (29.8, 39.7)
Southern	Mazabuka	35	20 (9.1, 37.5)												
Northern	Kasama	165	15.2 (10.2, 21.8)											142	80.3 (72.6, 86.3)
Eastern	Chadiza	37	29.7 (16.4, 47.2)												
	Chipata	100	67 (56.8, 75.9)			126	92.1 (85.5, 95.9)	115	81.7 (73.2, 88.1)	107	100 (95.7, 100)			300	80.3 (75.3, 84.6)
	Katete	204	68.6 (61.7, 74.8)	111	82.9 (74.3, 89.1)	176	54.5 (46.9, 62.0)	467	45.4 (40.8, 50.0)	204	100 (97.7, 100)	225	100 (97.9, 100)	318	68.9 (63.4, 73.9)
***An. gambiae s.l.***															
North-Western	Solwezi													20	100 (80.0, 100)
Copperbelt	Chililabombwe					45	71.1 (55.5, 83.2)			45	57.8 (42.2, 72.0)				
	Kitwe	464	38.4 (33.9, 43.0)	118	34.7 (26.4, 44.1)	42	11.9 (4.5, 26.4)	101	5.0 (1.8, 11.7)	134	3.7 (1.4, 8.9)	585	99.8 (98.9, 100)	643	98.8 (97.5, 99.4)
	Luanshya	35	54.3 (36.9, 70.8)			45	66.7 (51.0, 79.6)	100	51.0 (40.9, 61.1)	140	10.7 (6.3, 17.3)	10	100 (65.6, 100)	10	100 (65.6, 100)
	Mufulira													117	99.1 (94.6, 100)
	Ndola	40	82.5 (66.6, 92.1)									27	100 (84.5, 100)	254	98.8 (96.3, 100)
Northern	Kasama	80	38.8 (28.3, 50.3)											24	83.3 (61.8, 94.5)

**Table 2:** WHO bioassay test results on 2–5 day old adult mosquitoes from May 2012-April 2013 from 19 districts in Zambia.

Location																			
Province	District	Deltamethrin (0.05%)		Permethrin (0.75%)		λ-cyhalothrin (0.05%)		Etofenprox (0.50%)		DDT (4%)		Malathion (5%)		Pirimiphos-methyl (0.25%)		Bendiocarb (0.01%)		Propoxur (0.10%)	
		n	% mortality (95%CI)	n	% mortality (95%CI)	n	% mortality (95%CI)	n	% mortality (95%CI)	n	% mortality (95%CI)	n	% mortality (95%CI)	n	% mortality (95%CI)	n	% mortality (95%CI)	n	% mortality (95%CI)
***An. funestus s.l.***																			
North-Western	Chavuma	82	67.1 (55.7, 76.8)			26	57.7 (37.2, 76.0)							85	100 (94.6,100)	81	95.1 (87.2, 98.4)		
	Kabompo	101	89.1 (81.0, 94.2)									88	100 (94.8, 100)			213	100 (97.8, 100)		
	Mufumbwe	176	91.5 (86.1, 95.0)							75	100 (93.9, 100)	202	100 (97.7, 100)			251	100 (98.1, 100)		
	Mwinilunga	25	88.0 (67.7, 96.9)							24	100 (82.8, 100)	80	100 (94.3, 100)						
	Zambezi	52	71.2 (56.7, 82.5)			28	64.3 (44.1, 80.7)							129	100 (96.4, 100)	89	95.5 (88.3, 98.6)		
Western	Kaoma			158	68.4 (60.4, 75.4)					203	92.1 (873, 95.3)	127	100 (96.3, 100)			102	100 (95.5, 100)		
	Senanga															90	100 (94.9, 100)		
Central	Kapiri Mposhi															25	100 (83.4, 100)		
Lusaka	Luangwa					14	0 (0, 26.8)			56	92.9 (81.9, 97.7)								
Southern	Gwembe	60	41.2 (29.3, 55.1)			75	18.7 (10.9, 29.7)							42	100 (89.6, 100)	37	89.2 (73.6, 96.5)		
Luapula	Kawambwa	125	75.2 (66.5, 82.3)													136	14.0 (8.8, 21.2)		
	Mansa															20	0 (0, 20.1)		
Northern	Kasama	41	17.1 (7.7, 32.6)					138	5.1 (2.2, 10.6)										
Muchinga	Isoka													16	100 (75.9, 100)	62	8.1 (3.0, 18.5)		
	Mpika													22	100 (81.5, 100)				
Eastern	Chipata															18	44.4 (22.4, 68.7)		
***An. gambiae s.l.***																			
Copperbelt	Chililabombwe	61	85.2 (73.3, 92.6)							17	0 (0, 22.9)								
	Masaiti	102	64.7 (54.6, 73.7)			123	40.7 (32.0, 49.9)					98	100 (95.3, 100)	411	97.6 (95.4, 98.8)	119	92.4 (85.7, 96.3)	114	100 (95.9, 100)
	Mufulira	129	81.4 (73.4, 87.5)					111	86.5 (78.4, 92.0)	61	1.6 (0.1, 10.0)								
Muchinga	Isoka	50	94.0 (82.5, 98.4)																
	Mpika													65	100 (93.1, 100)				

*An. funestus s.s.* was resistant to pyrethroids throughout its range. Mortality to deltamethrin ranged from 15–76% in 2011–2012 and 17–91% in 2012–2013 (one population was potentially resistant in Mufumbwe District). Potential resistance to DDT was detected in Luangwa and Kaoma Districts in both years. Pyrethroid resistance was often accompanied by resistance to carbamates. However, this pattern was not seen in populations from North-Western and Western Provinces. Populations in this area were largely resistant to deltamethrin with mortality ranging from 67–91%, but susceptible or only potentially resistant to bendiocarb. The percent mortality to deltamethrin in populations from North-western and Western provinces was significantly greater than that in the rest of the country (81% vs. 48%, p<0.0001). All *An. funestus s.s.* populations were susceptible to organophosphates (Tables 1 and 2, Figures 2 and 3).

### Target site mutations

Sixty-seven *An. gambiae s.s.* were assayed for target site mutations that confer resistance from Kitwe and Kasama Districts (Copperbelt and Northern Provinces). The frequency of the *kdr* west allele (1014F) was 91%, but we did not detect *kdr* east (1014S) or *iACE* (Table 3).

**Table 3:** Genotypes of the voltage-gated sodium channel and acetylcholinesterase in *An. gambiae s.s.* from two locations in Zambia.

District	*kdr*			*iACE*		
	FF	LF	LL	rr	rs	ss
Kitwe	41	0	0	0	0	41
Kasama	16	8	2	0	0	25

F indicates *kdr* west allele (1014F), L is the susceptible. r is a resistant allele for the *Ace-1^R^* mutation (G119S), and s is susceptible.

### Metabolic Resistance Mechanisms

As expected, a large number of genes were significantly differentially expressed when comparing the field populations to the lab susceptible strains (Tables S3, S4, S5, S6, S7, S8). Where mosquito numbers permitted, an RC comparison was done (Kaoma λ-cyhalothrin resistant vs. unexposed and Luangwa etofenprox resistant vs. unexposed), and no significantly over expressed genes were found. This is likely because the RC arrays were comparing two genetically very similar groups, as resistance levels in both populations were high (Kaoma 31% mortality and Luangwa 30% mortality).

Genes associated with metabolic resistance (cytochrome P450s and glutathione S-transferases GSTs) were found over expressed in all six localities with microarray data (Table 4). The cytochrome P450 *CYP6Z3* was found to be over expressed in all localities except Solwezi. *CYP6M7* (ortholog of *CYP6M3* in *An. gambiae s.s.*) was over expressed in all localities. *CYP6P9a*, a gene which has been strongly associated with insecticide resistance in *An. funestus s.s.*, was observed in both the Katete (FC6.93) and Solwezi (FC3.10) localities.

**Table 4:** Over expressed annotated genes from gene families involved in detoxification in six vector populations in Zambia according to microarray (FC>2 and corrected p<0.05).

District	Gene Class	Gene	Fold change microarray	Fold change qRT PCR
***An. funestus***				
**Katete**	P450	*CYP6P9a*	6.93	403.57*
	P450	*CYP6M7*	4.16	6.08 (p-value = 0.06)
	P450	*CYP6Z3*	3.20	3.40*
	P450	*CYP6Z1*	3.00	13.31 (p-value = 0.09)
**Kaoma**	P450	*CYP6M7*	5.71	3.52*
	P450	*CYP6M4*	3.81	
	P450	*CYP6Z3*	2.69	2.99*
	P450	*CYP6S1*	2.06	
**Luangwa**	P450	*CYP6M7*	6.89	4.77*
	P450	*CYP6Y2*	2.78	
	P450	*CYP6S1*	2.3	
	P450	*CYP6Z3*	2.26	2.50*
**Solwezi**	P450	*CYP6M7*	3.19	3.73*
	P450	*CYP6P9a*	3.10	64.01*
	P450	*CYP4J9*	2.36	
**Kasama**	P450	*CYP6M7*	5.86	1.20
	P450	*CYP6M4*	3.67	
	P450	*CYP6Z3*	3.55	1.23
	P450	*CYP6Z1*	3.43	3.62
**Kabompo**	P450	*CYP6P9a*	nd	20.5*
	P450	*CYP6M7*	nd	2.6*
**Mufumbwe**	P450	*CYP6P9a*	nd	51.6*
	P450	*CYP6M7*	nd	6*
***An. gambiae***				
**Kitwe**	P450	*CYP6Z3*	12.04	3.76*
	P450	*CYP9K1*	3.19	
	P450	*CYP6M3*	2.93	
	P450	*CYP6AA1*	2.87	1.90*
	P450	*CYP4H24*	2.83	
	P450	*CYP9J4*	2.25	
	P450	*CYP306A1*	2.12	
	GST	*GSTE4*	6.88	2.05*
	GST	*GSTE1*	6.61	
	GST	*GSTD1_4*	2.65	
	GST	*GSTE3*	2.25	
	AChE	*Ace2*	2.25	
	Carboxylesterase	*COEAE1D*	2.50	3.06*

qRT PCR fold change values are presented where available.

*significantly different than susceptible strain.

nd not done.

**Figure 4:**
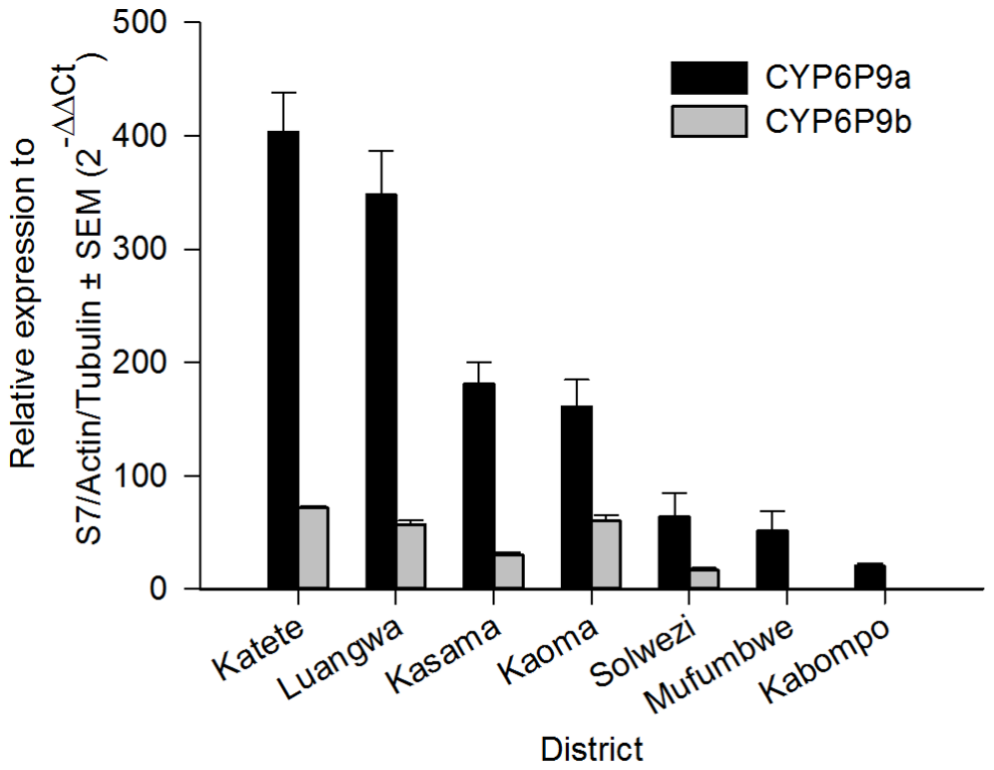
Differential expression by qRT PCR of *CYP6P9a* and *CYP6P9b* in *An. funestus* from 7 districts in Zambia. *CYP6P9b* data was not available for Mufumbwe and Kabompo.

**Figure 5:**
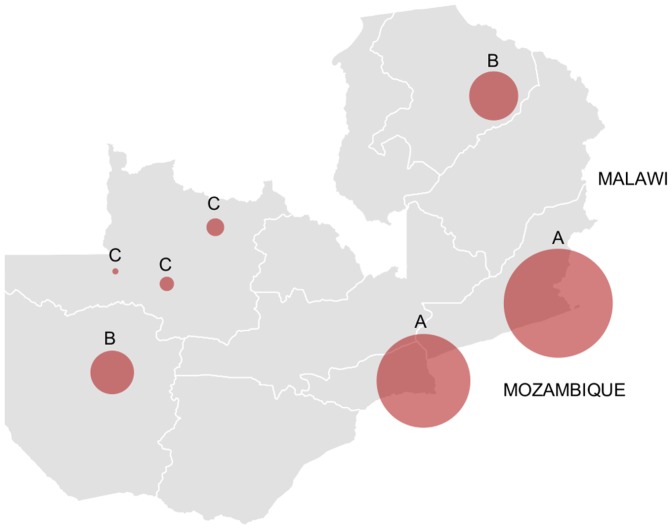
Over expression of *CYP6P9a* in *An. funestus* according to qRT PCR. The size of the circle represents the relative levels of over expression between populations. Circles bearing the same letters do not have significantly different fold-changes using student's t-test and an alpha of 0.05.

Four genes each in *An. funestus* and *An. gambiae* over expressed according to microarray were validated with qRT PCR. In all cases, FC values were not significantly different between C and R populations, so biological replicates were combined in these groups for each site. qRT PCR confirmed significant over expression in 16 out of 21 comparisons (Table 4). qRT PCR also showed significant over expression of *CYP6P9a* and *CYP6P9b* in all *An. funestus* populations (Figure 4), some of which were not revealed by microarray. There was a pattern of higher *CYP6P9a* expression closer to the Malawian and Mozambican borders than further away (Figure 5).

### Parasite prevalence

We detected a high prevalence of Plasmodium DNA in the head/thorax of wild caught mosquitoes after laying eggs (Table 5). The highest prevalence was 31.7% *P. falciparum* +ve in *An. gambiae* from Kitwe District.

**Table 5:** Prevalence of Plasmodium DNA in the heads/thoraces of wild caught F0 mosquitoes after being held to lay eggs.

District	Number tested	Number pf +ve	Number povm +ve	Number mixed pfovm +ve	Percent +ve
***An. funestus***					
Chipata	72	7			9.72
Gwembe	20	2			10.00
Kaoma	160	30	1	1	20.00
Kasama	44	5	2	1	18.18
Katete	13	0			0.00
Kawambwa	44	4			9.09
Luangwa	67	4			5.97
***An. gambiae***					
Kitwe	41	13			31.71
Kasama	30	3			10.00

The assay does not discriminate between *P. ovale*, *P. vivax*, and *P. malariae*.

## Discussion

Vector control was reintroduced as the frontline method of malaria prevention in Zambia in 2000, and since that time, has been rapidly scaled up to cover the entire country [17]. As in many countries, vector control with IRS and ITNs has relied almost entirely on pyrethroids and DDT. Pyrethroids are the only class of insecticides recommended for use on ITNs [35], and due to their low cost, relatively low mammalian toxicity, and long residual activity, they have also been extensively used by IRS programmes. Coinciding with increased use, there has been a rapid increase in reports of phenotypic resistance to these insecticides in sub-Saharan African Anopheles [8], [36], with Zambia reporting resistance in 2010 [16]. This prompted a rapid scale-up of entomological monitoring, and the formation of an insecticide resistance management technical working group to support the development of a well-informed insecticide resistance management plan.

Increased vector population monitoring through bioassays revealed that in *An. gambiae s.s.*, pyrethroid resistance is ubiquitous and is always accompanied by resistance to DDT, confirming a prior report of this resistance profile in the central part of Zambia [16]. This is a similar profile to that seen in Uganda [37] and Kenya [38] in East Africa. However, this species appears to be susceptible to both insecticides in the most southern part of its range in Mozambique [39], [40]. Carbamate resistance in this species is present in many parts of West Africa [41]–[44], but this is the most southerly that carbamate resistance has been reported in *An. gambiae s.s.* All populations were susceptible to organophosphates. However, potential resistance in *An. gambiae s.s.* to pirimiphos-methyl was detected in Copperbelt Province in 2013. This warrants further investigation with additional bioassays, as the country is likely to rely more heavily on organophosphates for vector control in the future.

In *An. funestus s.s*., pyrethroid resistance is common and is usually accompanied by resistance to bendiocarb. This is the same resistance profile as in Mozambique [45] and Malawi [46]. In North-western and Western Provinces, however, resistance to bendiocarb was absent or unconfirmed. In addition, mortality rates to deltamethrin were higher in this area of the country, indicating relatively greater susceptibility to pyrethroids. Combined, this pattern of resistance in *An. funestus s.s*. may indicate that the mechanism underlying pyrethroid and carbamate resistance has recently spread to the western side of the country and is being selected for by extensive use of pyrethroids in IRS and LLINs. This conclusion is supported by the pattern of over expression of P450s involved in pyrethroid resistance in this area (discussed below). Although *An. funestus s.s.* was susceptible to DDT throughout most of the country, potential resistance was documented in two areas.

Interestingly, none of the *An. gambiae s.l.* captured in this study were subsequently identified as *An. arabiensis*. This is unusual, as *An. arabiensis* is the more widely distributed member of the *An. gambiae* complex in Zambia. If *An. arabiensis* is more exophilic than *An. gambiae s.s.*, this may partially explain why none were captured, as collections were entirely based on indoor resting mosquitoes. However, *An. arabiensis* has been caught with success resting indoors in Zambia before [47]. Alternatively, it may be that recent vector control efforts have had a significant impact on *An. arabiensis* and current density is low in many places.

In *An. gambiae s.s.*, the resistance profile is partially mediated by target-site mutations. The *kdr* west allele (1014F) was found at very high frequencies, and was fixed in one population. This allele confers cross-resistance to both pyrethroids and DDT, which share the same target site. If this allele becomes fixed, the potential fitness cost of carrying the allele in the absence of insecticide would no longer be effective, and susceptible alleles would not be able to spread through the population.

Target-site resistance alone may not result in operational failure of vector control [48]. However, in concert with metabolic resistance, it can be a major threat. In Benin, where pyrethroid resistance is conferred by both target-site and metabolic mechanisms, sleeping under an ITN in an area with a resistant population provides little protection against being bitten [49]. In Zambia, metabolic resistance has been selected for in *An. gambiae s.s.* as well, involving an over expression of P450s involved in pyrethroid resistance and GSTs involved in DDT resistance. Of the P450s found over expressed in *An gambiae s.s.* from Zambia, *CYP6Z3*, *CYP6M3*, *CYP6AA1*, and *CYP4H24* have all been associated with other pyrethroid resistant populations in Africa [50]–[52], although none have yet been incriminated as insecticide metabolisers. Of the GSTs found over expressed in Zambia, *GSTE1*, *GSTE3*, and *GSTE4* have all been reported as elevated in a DDT resistant laboratory strain of *An. gambiae s.s.* originating from Tanzania [53].

Interestingly, *ace2* was over expressed in the *An. gambiae s.s.* population from Kitwe without the presence of an insensitive acetylcholinesterase (*iACE*) allele. Both *ace1* and *ace2* transcripts produce acetylcholinesterase, the target of carbamate and organophosphate insecticides. Although bioassays performed on this population showed susceptibility to carbamates and organophosphates, assays the next year on adjacent populations in Copperbelt Province indicated potential resistance. In *Aphis gossypii*, the *ace2* enzyme is significantly less sensitive to organophosphates than *ace1*, and a duplication in *ace2* was associated with organophosphate resistance [54]. This warrants further investigation in *An. gambiae s.s.*

In *An. funestus s.s.*, the resistance profile is mediated purely by metabolic mechanisms, namely an over expression of the P450s involved in pyrethroid metabolism. Although the incrimination of P450s in the metabolism of carbamates has yet to be shown directly, bioassays with piperonyl butoxide, an inhibitor of P450s, implicate this class as the causal mechanism behind carbamate resistance in *An. funestus s.s.* from southern Africa [55]. This mechanism may explain the cross-resistance seen between pyrethroids and carbamates in *An. funestus s.s*. from Zambia.

*CYP6P9a* was over expressed in all populations assessed by qRT PCR and has repeatedly been associated with pyrethroid resistance in *An. funestus s.s.* in southern Africa [56]–[59], and was recently found over expressed in Zambia [60]. It is able to metabolise both type I and type II pyrethroids [56]. A single allele of this gene appears to have swept through populations in Malawi and Mozambique, which indicates a single origin of this resistant phenotype [56]. Interestingly, expression of *CYP6P9a* in Zambia is highest in populations in the Southeast, perhaps indicating that resistance has arisen in this country from the known foci in Malawi and Mozambique.

DDT resistance appears to be emerging in *An. funestus s.s.* in the west and southern regions of Zambia. As target site resistance mechanisms have not been detected in *An. funestus s.s.*, it is likely that this resistance has a metabolic basis. Interestingly, several *CYP6Z* and *CYP6M* genes are over expressed in these populations and paralogues of these gene have been shown to metabolise DDT (and pyrethroids) [32], [61] in *An. gambiae s.s.* Further characterisation of these enzymes from *An. funestus s.s.* would be informative.

Extremely high levels of malaria infectivity were detected in this study, which is in contrast to previous findings of low infectivity of *An. funestus* and *An. gambiae* in IRS and ITN areas [16]. The vast majority of the specimens used in these assays (438/491) were collected in April or May of 2012, which coincides with the end of the rainy season. This may contribute to the high levels of infectivity seen in this study. Although sporozoite data collected here was not designed to measure entomological inoculation rates, the values are high enough to suggest that control is not effective. This requires further investigation if the control programme is to maintain goals and reduce incidence of the disease in Zambia further.

After the discovery of widespread resistance in the second half of 2011, an immediate shift in insecticide use for IRS was implemented in Zambia. The magnitude of this shift was restricted by the fact that insecticides had already been procured for the 2011 spray round. However, using the resistance data available at the time, it was decided that Northern, Muchinga, Luapula, and Copperbelt Provinces should be sprayed with bendiocarb, Eastern Province with organophosphates, and the rest of the country with pyrethroids. Simultaneously, a decision had to be made regarding which insecticides to procure for the 2012 spray round. With limited evidence at the time of extensive resistance in the west, a similar strategy was used in 2012. To better inform future decision-making, the following year (2012–2013) saw an increase in effort to document the resistance profile in North-Western and Western Provinces. As a result of this data acquisition, the National Malaria Control Centre is considering countrywide use of the organophosphate pirimiphos-methyl in 2013.

The resistance situation in the major malaria vectors in Zambia is worrying for the control programme. Because both metabolic and target-site mechanisms are underpinning the resistant phenotype, an operational significance of resistance to malaria control is likely. However, the impact of resistance on malaria transmission is an area that needs urgent investigation. Interestingly, a slight resurgence in malaria cases and deaths in Zambia has been documented between 2009 and 2011 [3], [15], although the causal mechanism is unknown. Since LLIN use is high, and pyrethroids are the only class of insecticides available for use in impregnated materials, the judicious use of pyrethroids for vector control is crucial to avoid operational failure. To this end, rotations or mosaic spraying of carbamates and organophosphates could be used for IRS, and pyrethroids only used for LLINs. Despite the higher cost of this strategy, it may be necessary in order to preserve the efficacy of currently available tools, and to make vector control a sustainable method of decreasing the burden of malaria. With proper management, the resistance gene frequency should reduce, and with continual monitoring, cheaper insecticides may be reintroduced in time.

In order to prevent insecticide resistance from compromising the sustainability of vector control, it is essential that good monitoring practices be established to enable early detection and appropriate response. Here, we have shown that an increased investment in monitoring and appropriate technical assistance have provided evidence to support informed decision-making. We demonstrate how modern techniques can quickly identify the genes involved in resistant malaria vectors and how that information can be used to develop an insecticide resistance management plan.

## Supporting Information

Table S1 **Locations of mosquito indoor resting collections between March 2011-April 2013.**
(DOCX)

Table S2 **Reference and candidate genes used in qRT PCR with primer sequences.**
(DOCX)

Table S3 **Genes demonstrating significant differential expression according to microarray between deltamethrin resistant** ***An. gambiae*** **from Kitwe and Kisumu susceptible strain.**
(XLSX)

Table S4 **Genes demonstrating significant differential expression according to microarray between Etofenprox resistant** ***An. funestus*** **from Katete and Fang susceptible strain.**
(XLSX)

Table S5 **Genes demonstrating significant differential expression according to microarray between Deltamethrin resistant** ***An. funestus*** **from Kasama and Fang susceptible strain.**
(XLSX)

Table S6 **Genes demonstrating significant differential expression according to microarray between unselected** ***An. funestus*** **from Solwezi and Fang susceptible strain.**
(XLSX)

Table S7 **Genes demonstrating significant differential expression according to microarray between Etofenprox resistant** ***An. funestus*** **from Luangwa and Fang susceptible strain.**
(XLSX)

Table S8 **Genes demonstrating significant differential expression according to microarray between λ-cyhalothrin resistant** ***An. funestus*** **from Kaoma and Fang susceptible strain.**
(XLSX)
