# Supplementary material for: Underpinning Sustainable Vector Control through Informed Insecticide Resistance Management
Source: PLoS One. 2014 Jun 16;9(6):e99822. doi: 10.1371/journal.pone.0099822 (PMC4059741; doi:10.1371/journal.pone.0099822)
Supplement: Table S1 — Locations of mosquito indoor resting collections between March 2011-April 2013. (DOCX) [file pone.0099822.s001.docx]

Table S1. Locations of mosquito indoor resting collections between March 2011-April 2013

| **District (coordinates)** | **Village** |
| --- | --- |
| Chadiza (32°36' E, 14°7' S) | Nsadzu |
| Chavuma (22°28' E, 13°12' S) | Kahuka |
| Chililabombwe (27°47' E, 12°20' S) | Kasapa |
|  | Kawama |
| Chipata (32°33' E, 13°37' S) | Chipungo |
|  | Mashanga |
| Gwembe (27°52' E, 16°36' S) | Makuyu |
|  | Munyumbwe |
| Isoka (33°17' E, 10°17' S) | Chiwanda |
|  | Londamaka |
|  | Malekani |
| Kabompo (24°19' E, 13°18' S) | Mubang'a |
|  | Mufuliwanjamba |
| Kaoma (24°53' E, 15°2' S) | Cheleka |
|  | Chilombo |
|  | Kafuta |
|  | Kapupa |
|  | Mangango |
|  | Namaloba |
| Kapiri Mposhi (28°24' E, 14°5' S) | Lukomba |
| Kasama (31° 5' E, 10°26' S) | Mponda |
|  | Nakapampa |
|  | Tibi |
| Katete (31°58' E, 14°7' S) | Mbinga |
|  | Undi |
|  | Unknown |
| Kawambwa (29°22' E, 9°53' S) | Kabanda |
|  | Mukamba |
| Kitwe (28°20' E, 12°46' S) | Chipata Compound |
|  | Kamatipa |
|  | Ndeke |
| Luangwa (29°56' E, 15°22' S) | Chilimanga |
| Luanshya (28°24' E, 13°3' S) | Walale |
| Mansa (29°8' E, 11°7' S) | Monga |
| Masaiti (28°47' E, 13°23' S) | Chishibambwe |
|  | Kafukanya |
| Mazabuka (27°59' E, 15°55' S) | Moobe |
| Mpika (31°28' E, 12°6' S) | Chalabesa |
|  | Mpepo |
| Mufulira (28°20' E, 12°35' S) | Kawama East |
|  | Mokambo |
| Mufumbwe (25°18' E, 13°54' S) | Matushi |
|  | Munyambala |
| Mwinilunga (24°50' E, 11°58' S) | Chibwika |
|  | Kabanda |
| Ndola (28°35' E, 12°58' S) | Chifubu |
|  | Chipulukusu |
|  | Kawama |
|  | Mushili |
| Senanga (23°50' E, 16°6' S) | Katula |
|  | Sikumbi |
| Solwezi (26°19' E, 12°24' S) | Kyabankaka |
| Zambezi (23°9' E, 13°36' S) | Chimuli |
|  | Kabambi |
