# Supplementary material for: Underpinning Sustainable Vector Control through Informed Insecticide Resistance Management
Source: PLoS One. 2014 Jun 16;9(6):e99822. doi: 10.1371/journal.pone.0099822 (PMC4059741; doi:10.1371/journal.pone.0099822)
Supplement: Table S2 — Reference and candidate genes used in qRT PCR with primer sequences. (DOCX) [file pone.0099822.s002.docx]

Table S2. Reference and candidate genes used in qRT PCR with primer sequences.

| **Gene** | **Species** | **Forward primer** | **Reverse primer** |
| --- | --- | --- | --- |
| **Reference genes** | | | |
| S7 | AG | CCTCTCGCTCTTTTTCCGGGCAT | CCTTGGAACCGAACACCATAGCGA |
| S7 | AF | GTGTTCGGTTCCAAGGTGAT | TCCGAGTTCATTTCCAGCTC |
| Elongation factor | AG | GGCAAGAGGCATAACGATCAATGCG | GTCCATCTGCGACGCTCCGG |
| Tubulin | AF | TCGGTAATGCCTGTTGGGAGCTGT | CCGCCGATGGTCTTGTCCGACG |
| Actin | AF | ttaaacccaaaagccaatcg | accggatgcatacagtgaca |
| **Candidate genes** | | | |
| CYP6AA1 | AG | AGGTCATCAATGAGACACTTCGTA | TCGCTCCCTCCACCTTGTA |
| CYP6Z3 | AG | CCACGCAATTGCATTGGTCT | TATGTTCTACGCGCATGGGG |
| COEAE1D | AG | GTCGAACCAAGCGAGAAAGC | GGAGCATGTCCAGTGCTTCTT |
| CYP6P9a | AF | CAGCGCGTACACCAGATTGTGTAA | TCACAATTTTTCCACCTTCAAGTAATTACCCGC |
| CYP6P9b | AF | CAGCGCGTACACCAGATTGTGTAA | TTACACCTTTTCTACCTTCAAGTAATTACCCGC |
| CYP6M7 | AF | CTATCGCCTCAGGGTGGAT | CAGTCGTAACATTATAGCCAAACC |
| CYP6Z1 | AF | GGATTTCCGATGAGGATTGA | GCAGCGTACTTGATTTACGG |
| CYP6Z3 | AF | TTTACCCATGCGGATAGAGC | TGGGTTTCCTTTGTACTACACATC |

AG – *Anopheles gambiae*, AF – *Anopheles funestus*
